# Life expectancy of HIV-positive individuals on combination antiretroviral therapy in Canada

**DOI:** 10.1186/s12879-015-0969-x

**Published:** 2015-07-17

**Authors:** Sophie Patterson, Angela Cescon, Hasina Samji, Keith Chan, Wendy Zhang, Janet Raboud, Ann N. Burchell, Curtis Cooper, Marina B. Klein, Sean B. Rourke, Mona R. Loutfy, Nima Machouf, Julio S. G. Montaner, Chris Tsoukas, Robert S. Hogg, CANOC collaboration

**Affiliations:** British Columbia Centre for Excellence in HIV/AIDS, Vancouver, Canada; Faculty of Health Sciences, BLU 9512, Simon Fraser University, 8888 University Drive, Burnaby, BC V5A 1S6 Canada; Dalla Lana School of Public Health, University of Toronto, Toronto, Canada; Division of Infectious Diseases, University Health Network, Toronto, Canada; Ontario HIV Treatment Network, Toronto, Canada; The Ottawa Hospital Division of Infectious Diseases, University of Ottawa, Ottawa, Canada; Faculty of Medicine, McGill University, Montreal, Canada; The Montreal Chest Institute, McGill University Health Centre, Montreal, Canada; Faculty of Medicine, University of Toronto, Toronto, Canada; Maple Leaf Medical Clinic, Toronto, Canada; Women’s College Research Institute, Toronto, Canada; Clinique Medicale l’Actuel, Montreal, Canada; Faculty of Medicine, University of British Columbia, Vancouver, Canada; Northern Ontario School of Medicine, Sudbury, Ontario Canada

**Keywords:** Life expectancy, Mortality, HIV, Antiretroviral therapy, CANOC, Canada

## Abstract

**Background:**

We sought to evaluate life expectancy and mortality of HIV-positive individuals initiating combination antiretroviral therapy (ART) across Canada, and to consider the potential error introduced by participant loss to follow-up (LTFU).

**Methods:**

Our study used data from the Canadian Observational Cohort (CANOC) collaboration, including HIV-positive individuals aged ≥18 years who initiated ART on or after January 1, 2000. The CANOC collaboration collates data from eight sites in British Columbia, Ontario, and Quebec. We computed abridged life-tables and remaining life expectancies at age 20 and compared outcomes by calendar period and patient characteristics at treatment initiation. To correct for potential underreporting of mortality due to participant LTFU, we conservatively estimated 30 % mortality among participants lost to follow-up.

**Results:**

9997 individuals contributed 49,589 person-years and 830 deaths for a crude mortality rate of 16.7 [standard error (SE) 0.6] per 1000 person-years. When assigning death to 30 % of participants lost to follow-up, we estimated 1170 deaths and a mortality rate of 23.6 [SE 0.7] per 1000 person-years. The crude overall life expectancy at age 20 was 45.2 [SE 0.7] and 37.5 [SE 0.6] years after adjusting for LTFU. In the LTFU-adjusted analysis, lower life expectancy at age 20 was observed for women compared to men (32.4 [SE 1.1] vs. 39.2 [SE 0.7] years), for participants with injection drug use (IDU) history compared to those without IDU history (23.9 [SE 1.0] vs. 52.3 [SE 0.8] years), for participants reporting Aboriginal ancestry compared to those with no Aboriginal ancestry (17.7 [SE 1.5] vs. 51.2 [SE 1.0] years), and for participants with CD4 count <350 cells/μL compared to CD4 count ≥350 cells/μL at treatment initiation (36.3 [SE 0.7] vs. 43.5 [SE 1.3] years). Life expectancy at age 20 in the calendar period 2000–2003 was lower than in periods 2004–2007 and 2008–2012 in the LTFU-adjusted analyses (30.8 [SE 0.9] vs. 38.6 [SE 1.0] and 54.2 [SE 1.4]).

**Conclusions:**

Life expectancy and mortality for HIV-positive individuals receiving ART differ by calendar period and patient characteristics at treatment initiation. Failure to consider LTFU may result in underestimation of mortality rates and overestimation of life expectancy.

**Electronic supplementary material:**

The online version of this article (doi:10.1186/s12879-015-0969-x) contains supplementary material, which is available to authorized users.

## Background

Twenty-five years since the World Health Organization announced the Global Program on AIDS to respond to the HIV/AIDS pandemic, an HIV cure remains elusive [1]. Recent reports estimate that 35 million people live with HIV globally, and this figure continues to increase [2]. That being said, treatment options available to individuals living with HIV have significantly improved in efficacy, safety and tolerability over the last decade. There is global consensus that combination antiretroviral therapy (ART) decreases mortality and morbidity among the HIV-positive population [3–5]; and new approaches in ART delivery, such as PI boosting and fixed-dose combinations, are thought to improve treatment outcomes [6]. The improving efficacy of ART regimens has resulted in the recognition of HIV as a chronic, manageable condition.

At the end of 2011 there were approximately 71,300 people living with HIV/AIDS in Canada [7]. Antiretroviral regimens have been available to Canadian residents eligible for treatment since the mid-1980s. Treatment provision and coverage vary across Canada, depending on the provincial and territorial programs implemented. With improvements in treatment regimen access, uptake and efficacy, the mortality and morbidity of HIV-positive persons have significantly decreased over time [8, 9]. However, despite widespread availability of more efficacious ART regimens, life expectancy (an important population health indicator) remains lower for HIV-positive individuals compared with the general population [8]. Additionally, other non-AIDS defining comorbidities are of increasing concern for HIV-positive individuals accessing ART; including malignancy, cardiovascular disease, pulmonary disease, liver disease, and renal disease [10, 11]. These comorbidities are hypothesized to occur at a higher rate among people living with HIV due to immunodeficiency [10], inflammation [12], a higher prevalence of behavioural risk factors [10], viral co-infections and the toxicity of antiretroviral regimens [13].

The effect of HIV on life expectancy in the era of ART is not well explored in the Canadian context. Similarly, the effect of baseline characteristics, such as sex and HIV transmission risk group, on life expectancy and mortality among treatment-experienced individuals has not yet been defined for our region. This analysis sought to evaluate life expectancy and mortality rates of HIV-positive individuals accessing antiretroviral regimens across three Canadian provinces, comparing outcomes in the modern treatment era by calendar year and by subgroups defined by patient characteristics at treatment initiation. In addition, we sought to consider the potential error introduced in mortality analyses due to loss to follow-up (LTFU) of cohort participants.

## Methods

### Data source

The Canadian Observational Cohort (CANOC) collaboration is a pan-provincial cohort of HIV-positive individuals initiating ART naively, established to evaluate patterns of treatment uptake and response, and health service provision and outcomes across Canada. CANOC consists of eight cohorts from the provinces of British Columbia, Ontario and Quebec. Each cohort site performs data extraction of demographic, laboratory and clinical variables of interest and annually submits these data to the coordinating centre in Vancouver, British Columbia. A detailed CANOC profile was published in 2011 [14].

### Ethics statement

Ethics board approval of the CANOC collaboration was granted by the Simon Fraser University Research Ethics Board (REB) and the University of British Columbia REB. Additionally, approval from local institutional review boards (IRBs) was granted at each participating cohort site, including: Providence Health Care Research Institute Office of Research Services, the Ottawa Hospital REB, University Health Network (UHN) REB, Véritas IRB, Biomedical C REB of the McGill University Heath Centre (MUHC), University of Toronto HIV REB, and Women’s College Hospital REB.

Written consent for study participation has been obtained from all study cohorts except: HAART Observational Medical Evaluation and Research Cohort (IRB approves the retrospective use of anonymous administrative data without requiring consent; an information sheet is provided in lieu of a consent form); Ottawa Hospital Cohort, UHN and Maple Leaf Medical Clinic (IRB/REBs approve the anonymous use of data retrospectively extracted from clinical care databases without requiring consent); and MUHC and the Electronic Antiretroviral Therapy Cohort (IRB/REBs approve the anonymous use of data retrospectively extracted from clinical care databases without requiring consent; patients sign a general waiver on opening a medical chart at the hospital but no specific study related consent).

### Inclusion criteria

CANOC includes individuals with documented HIV infection, aged at least 18 years, and currently resident in Canada. Participation is restricted to formerly ART-naive HIV-positive individuals initiating combination ART on or after 1 January 2000. All participants had baseline CD4 cell count and viral load measurements (within six months prior to ART initiation). This analysis included individuals enrolled in CANOC from 2000–2012.

### Outcomes and statistical methods

The primary outcome of interest was date of death from all-causes, used to assess mortality rates and life expectancy. Demographic and clinical characteristics of interest included sex, Aboriginal ancestry, baseline CD4 cell count and HIV transmission group (injection drug use (IDU) history vs. no IDU history).

This analysis compared life expectancy among HIV-positive individuals on ART in Canada over three calendar periods, based on year of ART initiation (2000–2003, 2004–2007 and 2008–2012). We also considered differences in mortality rates and life expectancy in subgroups defined by patient characteristics at initiation of treatment. Crude and age-specific mortality rates were calculated. Mortality rates (per 1000 person-years) were calculated by dividing the total number of deaths by the total number of person-years of follow-up; we report these results with standard errors, calculated using Poisson distribution. Mortality rates were stratified by sex (male vs. female), Aboriginal ancestry (Aboriginal vs. non-Aboriginal), HIV transmission risk group (IDU history vs. no IDU history), and baseline CD4 count (<350 and ≥350 cells/μL). Where the data set was incomplete, analyses were restricted to cohort participants with non-missing information.

Abridged life tables were constructed from age-specific mortality rates to compare life expectancy at the age of 20 years in the three designated calendar periods under study. Life expectancy at age 20 refers to the number of remaining years a participant would be expected to live. Abridged life tables constructed for this analysis were based on widely used and standardized methods [15]. We partitioned each individual’s total person-time contribution and deaths into five-year age categories to compute abridged life tables and life expectancies with corresponding standard errors [SE]. Due to the relatively small proportion of participants within older age categories, the final age category constructed within the life tables was 55+ years. Life expectancy values at the exact age of 20 years were reported for the whole cohort as well as stratified by sex, transmission group, Aboriginal ancestry and CD4 cell count at treatment initiation.

### Estimation of potential error due to LTFU

For the purposes of this analysis, LTFU was defined as the last recorded known contact date being more than 18 months before the end of the study period. To correct for potential misclassification of death among persons recorded as lost to follow-up (and differences in ascertainment rates between individual cohorts/provinces), we conservatively estimated that 30 % of participants who were lost to follow-up had died. This estimate was recently used in a mortality analysis conducted within the Antiretroviral Therapy Cohort Collaboration (ART-CC) [16], chosen to reflect the findings of a survey evaluating mortality among those lost to follow-up within the French hospital database on HIV infection (FHDH) [16, 17]. Our 30% estimate is consistent with published literature describing mortality rates among HIV-positive participants lost to follow-up in a Ugandan setting, ranging from 28–29 % [18–20], and Canadian estimates of mortality rates among HIV-hepatitis C co-infected participants lost to follow-up, reported at 40 % [21].

Among participants not lost to follow-up, a multivariable logistic regression model (containing demographic and clinical characteristics) was created, with death as the outcome variable. The resulting parameter estimates were then applied to those lost to follow-up, which generated estimated probabilities of death for these lost patients, of which the highest 30 % were categorized as deaths. Date of death was recorded as the last contact date for participants lost to follow-up who were assigned the outcome.

## Results

Baseline characteristics of the 9997 participants within the analytic sample are presented in Table 1. The cohort was predominantly male (82 %), with a history of IDU reported by 27 % of participants. The median baseline age was 40 (interquartile range (IQR): 33–47), with 10 % of participants reporting Aboriginal ancestry. Of the 9350 participants with available data on hepatitis C status, 26 % were hepatitis C positive. Notably, among participants with a history of IDU the prevalence of hepatitis C infection was 82 %, compared to 7 % among participants with no IDU history (*p* < 0.001).

**Table 1:** Demographic and clinical characteristics of participants, overall and by calendar year of ART initiation (*n* = 9997)

	Period 1	Period 2	Period 3	Overall
	(2000–2003)	(2004–2007)	(2008–2012)	(2000–2012)
	*n* = 2381	*n* = 3058	*n* = 4558	*n* = 9997
	(%)	(%)	(%)	(%)
Baseline age				
18–34	693 (29.1)	791 (25.9)	1459 (32.0)	2943 (29.4)
35–44	1034 (43.4)	1297 (42.4)	1518 (33.3)	3849 (38.5)
45–54	481 (20.2)	718 (23.5)	1145 (25.1)	2344 (23.4)
55+	173 (7.3)	252 (8.2)	436 (9.6)	861 (8.6)
Sex				
Female	480 (20.2)	591 (19.3)	760 (16.7)	1831 (18.3)
Male	1901 (79.8)	2467 (80.7)	3798 (8303)	8166 (81.7)
History of IDU^a^				
Yes	592 (31.0)	719 (29.5)	830 (23.7)	2141 (27.3)
No	1315 (69.0)	1721 (70.5)	2666 (76.3)	5702 (72.7)
Aboriginal ancestry^b^				
Yes	150 (10.6)	151 (9.7)	166 (9.4)	467 (9.8)
No	1266 (89.4)	1410 (90.3)	1599 (90.6)	4275 (90.2)
CD4 cell count (cells/μL)				
<350	2024 (85.0)	2679 (87.6)	2986 (65.5)	7689 (76.9)
≥350	357 (15.0)	379 (12.4)	1572 (34.5)	2308 (23.1)

Analyses restricted to those with non-missing data

*IDU* injection drug use

^a^ *n* = 7843

^b^ *n* = 4742

### Loss to follow-up

The median follow-up time within the cohort was 52 months (IQR: 24–89). Overall, 11 % of all participants were lost to follow-up during the study period. Table 2 compares the clinical and sociodemographic characteristics at treatment initiation for participants retained within the cohort and participants lost to follow-up. Participants lost to follow-up were more likely to be female, report no IDU history, report no Aboriginal ancestry and have a CD4 count <350 cells/μL at treatment initiation, as compared to participants retained in the cohort (all p <0.05). Table 3 compares the clinical and sociodemographic characteristics at treatment initiation for participants lost to follow-up who were assigned and not assigned the outcome death. Participants assigned the outcome death were more likely to report IDU history and Aboriginal ancestry, and have a CD4 count <350 cells/μL at treatment initiation (all *p* < 0.001). In all analyses, adjusting for mortality among cohort participants lost to follow-up decreased the estimated life expectancy and increased the estimated mortality rate, as expected.

**Table 2:** Clinical and sociodemographic characteristics at treatment initiation for participants retained within the cohort and participants lost to follow-up (*n* = 9997)

Characteristic	Total (n)	Not lost to follow-up (%)	Lost to follow-up (%)	P-value
All Participants	9997	8865 (88.7)	1132 (11.3)	
Sex				0.044
Female	1831	1599 (87.3)	232 (12.7)	
Male	8166	7266 (89.0)	900 (11.0)	
History of IDU^a^				<0.001
Yes	2141	1964 (91.7)	177 (8.3)	
No	5702	5051 (88.6)	651 (11.4)	
Aboriginal ancestry^b^				0.001
Yes	467	437 (93.6)	30 (6.4)	
No	4275	3788 (88.6)	487 (11.4)	
CD4 cell count (cells/μL)				0.002
<350	7689	6777 (88.1)	912 (11.9)	
≥350	2308	2088 (90.5)	220 (9.5)	

*IDU* injection drug use

^a^ *n* = 7843

^b^ *n* = 4742

**Table 3:** Clinical and sociodemographic characteristics at treatment initiation for participants assigned to the outcome death and not assigned to death among those lost to follow-up (*n* = 1132)

Characteristic	Total (n)	Not assigned death (%)	Assigned death (%)	P-value
Participants lost to follow-up	1132	792 (70.0)	340 (30.0)	
Sex				0.787
Female	232	164 (70.7)	68 (29.3)	
Male	900	628 (69.8)	272 (30.2)	
History of IDU^a^				<0.001
Yes	177	53 (29.9)	124 (70.1)	
No	651	538 (82.6)	113 (17.4)	
Aboriginal ancestry^b^				<0.001
Yes	30	13 (43.3)	17 (56.7)	
No	487	379 (77.8)	108 (22.2)	
CD4 cell count (cells/μL)				<0.001
<350	912	604 (66.2)	308 (33.8)	
≥350	220	188 (85.5)	32 (14.5)	

*IDU* injection drug use

^a^ *n* = 828

^b^ *n* = 517

### Mortality

9997 individuals contributed 49,589 person-years and 830 deaths for an overall crude mortality rate of 16.74 [SE 0.58] per 1000 person-years (Table 4). After estimating 30 % mortality among participants lost to follow-up, there were 1170 deaths, with a mortality rate of 23.59 [SE 0.69] per 1000 person-years. Unadjusted and adjusted mortality rates, overall and by select characteristics, are presented in Table 4. In both unadjusted and LTFU adjusted analyses, female sex, IDU history, Aboriginal ancestry and CD4 count of <350 cells/μL at treatment initiation were significantly associated with increased mortality.

**Table 4:** Population size, deaths, and unadjusted and adjusted mortality rate per 1000 person-years (PY), overall and by select characteristics

	Population^a^	Deaths	Person Years (PY)	Unadjusted mortality rate per 1000PY (SE)	Adjusted^b^ mortality rate per 1000PY (SE)
Overall	9997	830	49588.88	16.74 (0.58)	23.59 (0.69)
Sex					
Female	1831	184	9303.73	19.78 (1.46)	27.09 (1.71)
Male	8166	646	40285.14	16.04 (0.63)	22.79 (0.75)
History of IDU					
Yes	2141	377	11082.71	34.02 (1.75)	45.21 (2.02)
No	5702	257	28875.05	8.90 (0.56)	12.81 (0.67)
Aboriginal ancestry					
Yes	467	123	2398.97	51.27 (4.62)	58.36 (4.93)
No	4275	255	24627.65	10.35 (0.65)	14.74 (0.77)
Baseline CD4 (cells/μL)					
<350	7689	740	41016.06	18.04 (0.66)	25.55 (0.79)
≥350	2308	90	8572.82	10.50 (1.12)	14.23 (1.29)
Year of ART initiation					
2000–2003	2381	399	20389.75	19.57 (0.98)	31.29 (1.24)
2004–2007	3058	287	17928.23	16.01 (0.95)	21.03 (1.08)
2008–2012	4558	144	11270.90	12.78 (1.07)	13.75 (1.11)

*SE* standard error, *IDU* injection drug use, *ART* antiretroviral therapy

^a^Analyses restricted to those with non-missing data

^b^Including assumed 30 % mortality in participants lost to follow-up

### Life expectancy

**Fig. 1:**
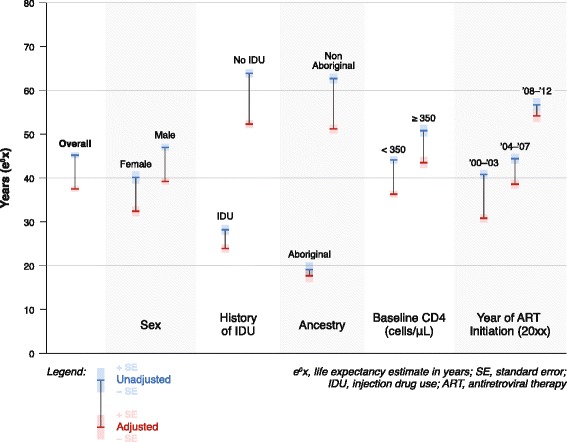
Life expectancy estimates at age 20 years, showing unadjusted and adjusted values, by clinical and sociodemographic characteristics at baseline

Unadjusted life expectancy at age 20 was 45.2 [SE 0.66], and 37.5 [SE 0.61] years after estimating 30 % mortality among those lost to follow-up (Table 5). In both unadjusted and LTFU adjusted analyses, female sex, IDU history, Aboriginal ancestry and CD4 count of <350 cells/μL at treatment initiation were significantly associated with decreased life expectancy at age 20 (Table 5, Fig. 1).

**Table 5:** Life expectancy estimates at age 20 years, showing unadjusted and adjusted values (assuming 30 % mortality among those lost to follow-up), n = 9997

	Unadjusted	Adjusted
	e^O^x (SE)	e^O^x (SE)
Overall	45.2 (0.66)	37.5 (0.61)
Sex		
Female	40.1 (1.34)	32.4 (1.12)
Male	47.0 (0.75)	39.2 (0.72)
History of IDU		
Yes	28.2 (1.07)	23.9 (0.96)
No	63.9 (0.92)	52.3 (0.84)
Aboriginal ancestry		
Yes	19.1 (1.60)	17.7 (1.49)
No	62.7 (1.12)	51.2 (0.97)
Baseline CD4 (cells/μL)		
<350	44.1 (0.77)	36.3 (0.70)
≥350	50.8 (1.35)	43.5 (1.25)
Year of ART initiation		
2000–2003	40.8 (1.05)	30.8 (0.90)
2004–2007	44.4 (1.04)	38.6 (0.96)
2008–2012	56.7 (1.43)	54.2 (1.37)

*e* ^*o*^ *x* life expectancy estimate (years), *SE* standard error, *IDU* injection drug use, *ART* antiretroviral therapy

### Temporal trends

Both life expectancy and mortality rates improved over time (Tables 4 and 5, Fig. 1). Mortality rates decreased within more recent time periods, both before and after adjusting for mortality among participants lost to follow-up (Table 4). Life expectancy at age 20 increased within more recent time periods in both the unadjusted and adjusted analyses, reaching a maximum life expectancy of 54.2 [SE 1.37] years in the period 2008–2012, after adjusting for mortality among participants lost to follow-up (Table 5).

### Sensitivity analysis

Given that only 8.6 % (*n* = 861) of our analytic sample was aged 55+ years (contributing a total of 3903.8 person-years of follow-up) we were concerned that mortality rates among participants within this age category, especially at the oldest ages, may be underestimated, potentially inflating the life expectancy estimates generated. To explore this issue, we conducted a sensitivity analysis using 2006 mortality data for the general Canadian population to obtain revised life expectancy estimates [22]. We assumed that the rise in the mortality rate between participants aged 50–54 and participants aged 55+ years in our analytic sample would increase by the same ratio as that demonstrated within the general Canadian population of corresponding age categories. Based on this assumption, we extrapolated the mortality rates observed among study participants aged 50–54 to produce new mortality estimates for participants aged 55+ years. After performing this adjustment, the overall life expectancy at age 20 decreased from 45.2 [SE 0.7] years to 31.7 [SE 0.4] years (Additional file 1).

## Discussion

The largest of its kind conducted in Canada to date, our study contributes a number of important findings on life expectancy among HIV-positive persons in this region. Specifically, we observed that life expectancy and mortality for HIV-positive individuals accessing ART differ by sex, IDU history, Aboriginal ancestry, baseline CD4 cell count prior to ART initiation, and calendar period of ART initiation. Additionally, this manuscript employs a novel methodological approach to life expectancy analyses; questioning the validity of cohort studies that fail to account for mortality among participants lost to follow-up and those that lack sufficient follow-up data among older individuals accessing treatment.

Life expectancy within this cohort improved with more recent calendar periods. This finding has been previously reported in the literature, and likely reflects improved coverage and quality of ART regimes and HIV care [4, 5, 23, 24]. Recent reports estimate the average remaining life expectancy for Canadians at age 20 to be 59.7 and 63.9 years for men and women, respectively [25]. When compared with the findings of our study, it is clear that despite advances in the health care services and antiretroviral therapies available to people living with HIV in Canada, the life expectancy of HIV-positive Canadians remains lower than that of the general population.

Men demonstrated longer life expectancy and lower mortality rates compared to women in this cohort. This is in contrast to population-level surveillance data published by Statistics Canada, reporting increased life expectancy among women compared with men in the general population in all Canadian provinces [26]. A recent publication evaluating regional and sex specific patterns of HIV/AIDS mortality in Canada from 1987-2008 found that age-specific death rates among women in most age categories were lower than those observed among men [27]. However, this study observed that the decline in HIV-related mortality rates since the mid-1990s was much more pronounced among men than women. North American publications evaluating mortality in HIV-positive cohorts accessing ART have demonstrated comparable sex-related differences to those shown in our study [9, 28, 29]. However, mortality analyses conducted in European and trans-continental settings have observed contrasting sex differences among HIV-positive cohorts, depicting a longer life expectancy among women than men [8, 30, 31]. Other studies have found no sex-related differences [32].

These inconsistencies may be due to inherent differences in cohort demographics, treatment access and healthcare utilization. A recent publication commented on the validity of cross-cohort comparisons of mortality rates, suggesting that cohort characteristics in different settings can significantly alter HIV outcomes, including mortality [16]. In recent years, Canadian studies have reported disparities in retention within key stages of the HIV treatment cascade, including access and adherence to treatment and achievement of viral suppression, according to region of care [33], sex and IDU history [33, 34]. In these studies, women and individuals with a history of IDU demonstrated decreased retention within the HIV treatment cascade in Canada [33, 34]. In CANOC, a disproportionate number of HIV-positive individuals reporting IDU history are women, which reflects the composition of the Canadian HIV epidemic, especially in British Columbia.

Individuals with a history of IDU demonstrated poor mortality and life expectancy outcomes in this analysis. This finding has been consistently reported in the published literature [8, 9, 23, 29, 35]. Previous studies have shown that the increased mortality rates among persons with IDU history primarily reflect higher rates of all-cause mortality, rather than HIV-related mortality [35, 36]. Indeed this demographic group experiences a high prevalence of comorbidities, augmented by potential active drug use, socioeconomic disadvantage and poor access to health care services [36]. In our study cohort, a significantly higher prevalence of hepatitis C infection was identified among participants with a history of IDU compared to individuals with no IDU history. Infection with hepatitis C is an important contributor to mortality and morbidity among people living with HIV, primarily due to an increased risk of liver disease [37, 38]. A recent Canadian cohort study reported notably higher mortality rates among individuals co-infected with HIV and hepatitis C compared to previously published mortality rates among mono-infected HIV-positive individuals [39].

CD4 cell count at treatment initiation was another significant predictor of mortality and life expectancy in our cohort. Participants with CD4 cell counts <350 cells/μL at treatment initiation demonstrated increased mortality rates and decreased life expectancy. This finding is consistent with previous mortality studies [8, 9], and reinforces the long-term health benefits of ART initiation earlier in the course of HIV infection. Earlier ART initiation is supported by the 2014 International Antiviral Society-USA guidelines, recommending the initiation of antiretroviral therapy regardless of CD4 cell count for most patients [40].

In our analysis, we found Aboriginal ancestry to be predictive of decreased life expectancy. This is consistent with a Canadian retrospective cohort study published in 2011, which reported that individuals of Aboriginal descent receiving ART demonstrate increased all-cause and HIV-related mortality rates compared with non-Aboriginal people living with HIV in Canada [35]. This observation may be due to competing life circumstances and social-structural factors that influence access and adherence to ART among Aboriginal persons [35]. A report by Statistics Canada similarly identified this difference in life expectancy between Canadians of Aboriginal ancestry and the non-Aboriginal population [41]. This observation is concerning, and initiatives to improve treatment outcomes of Aboriginal Canadians should be made a priority. To note, this finding may be influenced by underlying characteristics of our cohort, as a disproportionate number of HIV-positive individuals reporting IDU history in CANOC also report Aboriginal ancestry.

Large-scale longitudinal cohort studies often face a significant limitation in the form of incomplete data due to participant LTFU. LTFU affects most cohort studies and can result in bias, affecting study validity [42]. In conducting this study we sought to account for the impact of LTFU on life expectancy estimates to reduce potential bias and improve the validity of our findings. After conservatively estimating 30 % mortality among participants lost to follow-up, unsurprisingly we observed that mortality rates increased and life expectancy estimates decreased. This suggests that failure to account for participant lost to follow-up in mortality analyses results in the potential for significant underestimation of mortality rates and overestimation of life expectancy data, affecting data validity.

### Limitations

Several limitations of this study should be acknowledged. CANOC includes data from only three Canadian provinces, and participants included are already linked to specific health care facilities, therefore the analytic sample is not be fully representative of the overall HIV-positive population across Canada, including those most at risk of adverse clinical outcomes. Data from British Columbia include all CANOC-eligible HIV-positive individuals in the province accessing ART, whereas data from Ontario and Quebec are collected from a selection of clinics, which may introduce a clinic-selection bias. This study considers the Canadian HIV-positive population, therefore observations described may not be generalizable to other global settings.

We conducted an adjusted analysis to account for misclassification of mortality among participants lost to follow-up, conservatively estimating the death of 30 % of those lost to follow-up. As published data concerning mortality among participants lost to follow-up in Canadian cohorts are limited, this estimate was based on previous European [17] and Ugandan studies among HIV-positive cohorts [18–20] and findings from a Canadian cohort of HIV-hepatitis C co-infected individuals [21]. Therefore, this estimate may not be entirely generalisable to all HIV-positive individuals in Canada. Further studies may seek to evaluate how accurate our assumption was regarding mortality among HIV-positive participants lost to follow-up in this setting. Differences in mortailty ascertainment by site also exist, and thus our estimation of mortality may be more problemetic at sites that do not link to vital statistics registries.

Another potential limitation of this study was that we did not allow for censoring in LTFU data. This is particularly relevant for those who initiated ART in the most recent time period; in some cases not enough time had passed to be declared lost to follow-up given the 18-month definition. Unfortunately, data describing causes of death are not currently collected across participating cohorts in the CANOC collaboration; as such we were unable to present the distribution of causes of death within this manuscript.

Given that only a small proportion of our analytic sample was aged ≥55 years, we conducted a sensitivity analysis based on mortality rates within the general Canadian population to explore whether possible underestimation of mortality rates among participants within this age category affected the life expectancy values generated. The results suggested that our life expectancy estimates may be overestimated due to imprecise representation of mortality rates within older age categories. As the Canadian population living with HIV ages, the reliability of results generated by life expectancy analyses such as this will continue to improve.

## Conclusion

In conclusion, this study found that while both mortality rates and life expectancy for HIV-positive Canadians accessing ART are improving over time, they remain below that of the general Canadian population. Life expectancy and mortality are influenced by baseline characteristics at treatment initiation, including Aboriginal ancestry, sex, CD4 cell count and IDU history. These findings suggest the need for targeted interventions for patient subgroups at increased risk of adverse outcomes. Future studies examining patterns of life expectancy and mortality among HIV-positive populations should take into account mortality among participants lost to follow-up to ensure validity of estimates.

## Additional file

Additional file 1:
**Life expectancy estimates at age 20 years, showing unadjusted values, estimates adjusted for mortality among participants lost to follow-up, and estimates adjusted to account for low proportion of participants aged ≥55 years, based on 2006 Canadian mortality data (** ***n*** **= 9997).**
