# Supplementary material for: Life expectancy of HIV-positive individuals on combination antiretroviral therapy in Canada
Source: BMC Infect Dis. 2015 Jul 17;15:274. doi: 10.1186/s12879-015-0969-x (PMC4504463; doi:10.1186/s12879-015-0969-x)
Supplement: Additional file 1: — Life expectancy estimates at age 20 years, showing unadjusted values, estimates adjusted for mortality among participants lost to follow-up, and estimates adjusted to account for low proportion of participants aged ≥55 years, based on 2006 Canadian mortality data ( n = 9997). [file 12879_2015_969_MOESM1_ESM.doc]

**APPENDIX**

**Table 6:** Life expectancy estimates at age 20 years, showing unadjusted values, estimates adjusted for mortality among participants lost to follow-up, and estimates adjusted to account for low proportion of participants aged ≥55 years, based on 2006 Canadian mortality data (n=9997).

|  | **Unadjusted eox**  **[SE]** | **eox adjusted for LTFU [SE]** | **eox adjusted for participants aged**  **≥55** **[SE]** |
| --- | --- | --- | --- |
| Overall | 45.2 (0.66) | 37.5 (0.61) | 31.7 (0.39) |
| Male | 47.0 (0.75) | 39.2 (0.72) | 33.1 (0.45) |
| Female | 40.1 (1.34) | 32.4 (1.12) | 29.5 (0.78) |
| IDU | 28.2 (1.07) | 23.9 (0.96) | 23.8 (0.82) |
| Non-IDU | 63.9 (0.92) | 52.3 (0.84) | 38.8 (0.44) |
| Aboriginal | 19.1 (1.60) | 17.7 (1.49) | 18.2 (1.48) |
| Non-aboriginal | 62.7 (1.12) | 51.2 (0.97) | 36.6 (0.51) |
| CD4 <350 | 44.1 (0.77) | 36.3 (0.70) | 30.9 (0.46) |
| CD4 350+ | 50.8 (1.35) | 43.5 (1.25) | 38.0 (0.86) |
| 2000-2003 | 40.8 (1.05) | 30.8 (0.90) | 29.3 (0.64) |
| 2004-2007 | 44.4 (1.04) | 38.6 (0.96) | 32.3 (0.65) |
| 2008-2012 | 56.7 (1.43) | 52.4 (1.37) | 36.6 (0.73) |

eox: life expectancy estimate (years); SE: standard error; IDU: injection drug use; ART: antiretroviral therapy; LTFU: loss to follow-up.
